# Supplementary material for: Interactive effects of temperature and salinity on metabolism and activity of the copepod Tigriopus californicus
Source: J Exp Biol. 2024 Sep 6;227(17):jeb248040. doi: 10.1242/jeb.248040 (PMC11418200; doi:10.1242/jeb.248040)
Supplement: Supplementary information [file jexbio-227-248040-s1.pdf]

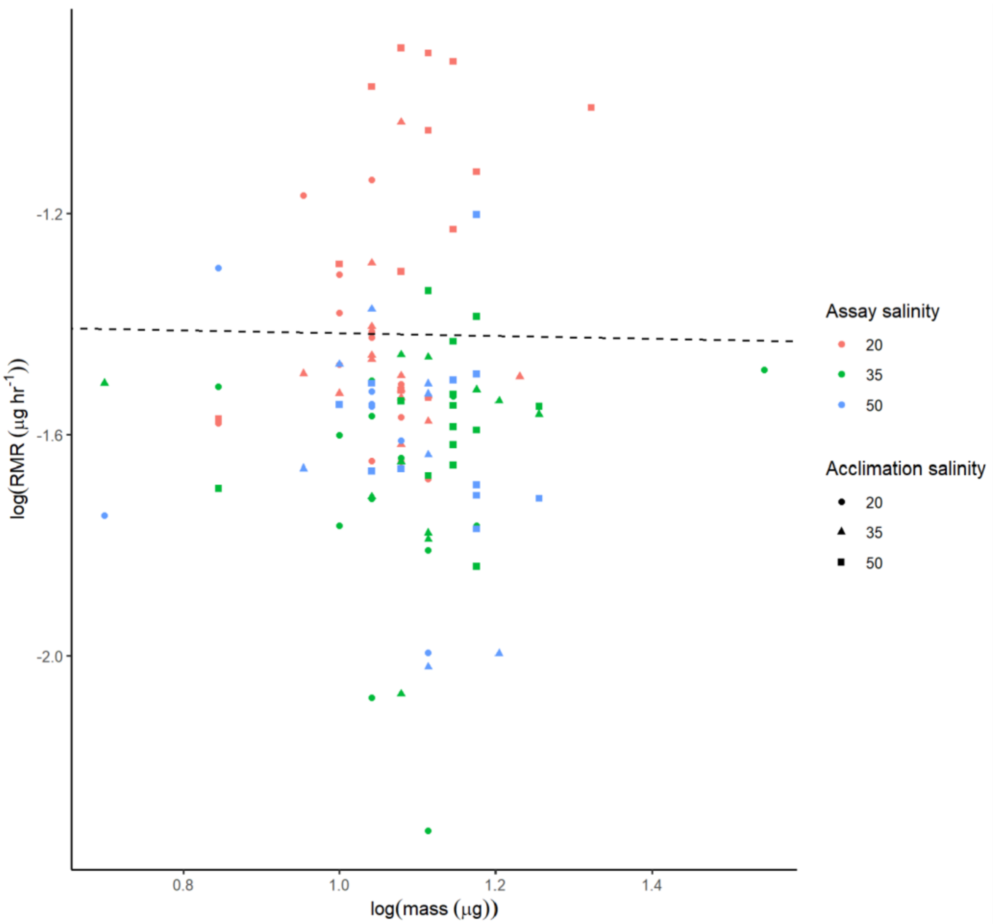

Fig. S1. Log of copepod metabolic rates under 17.5°C does not scale allometrically with the log of mass. Dashed line represents the slope and intercept from a general linear model (log(RMR) ~ acclimation salinity + assay salinity + log(mass)) examining the effect of mass on metabolic rate of copepods measured under 17.5°C. Point color represents assay salinity, and point shape represents acclimation salinity. Because of this observed lack of allometric scaling, the unreliability of measured masses of 27.5°C copepods, and the possible influences of inter-individual variation in activity levels on RMR, we chose to forego correcting copepod RMRs by mass in our analyses.

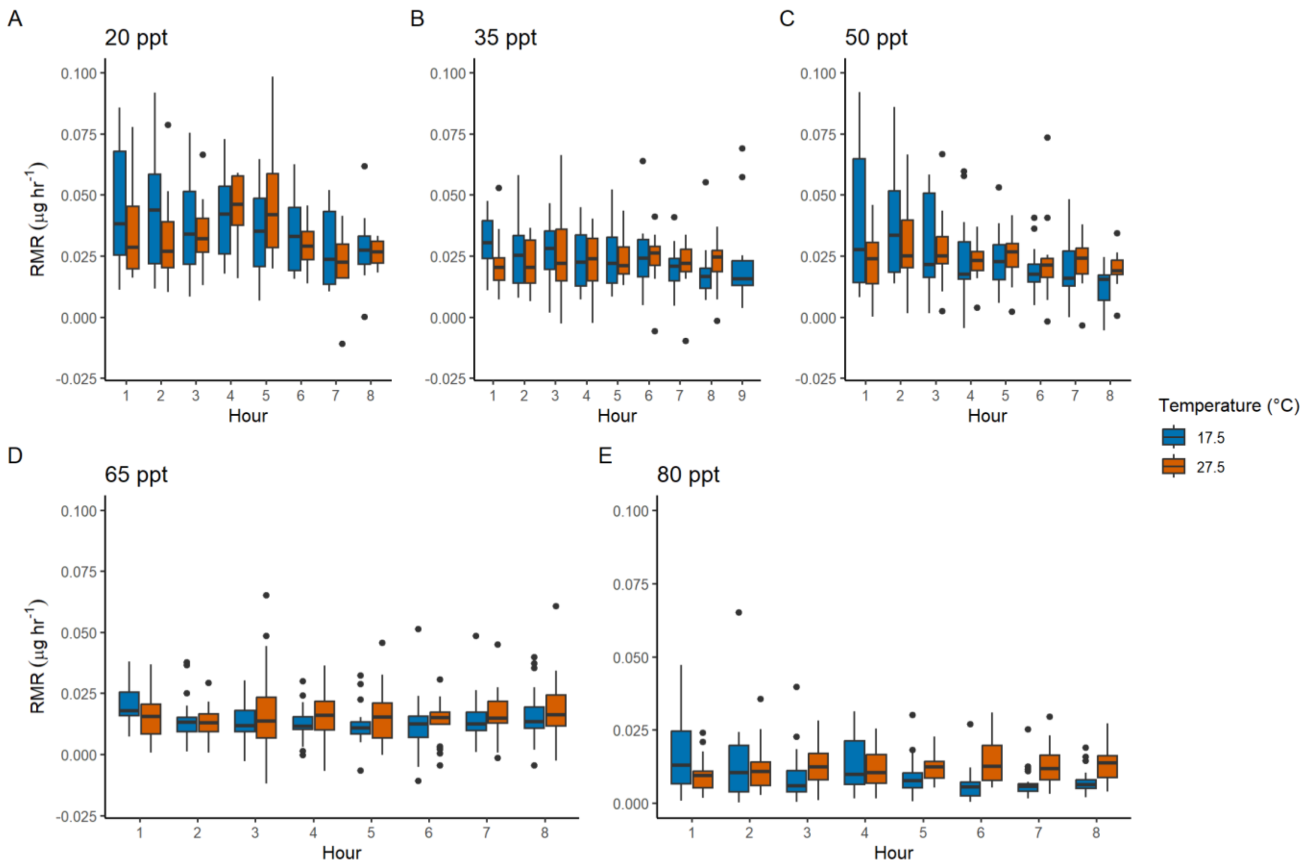

Fig. S2. Hourly measures of RMR in chronic acclimation copepods show no acute temperature response. Based on the Arrhenius relationship we would anticipate 2-3x higher rates at 27.5°C than at 17.5°C, at least initially. Box plots representing RMR of copepods chronically acclimated to 20, 35, 50, 65, and 80 ppt (A-E, respectively) measured over hour-long periods. Median values are depicted by black lines in the middle of boxes, upper and lower box hinges correspond to the first and third quartiles, and whiskers extend to no further than 1.5 times the interquartile range.

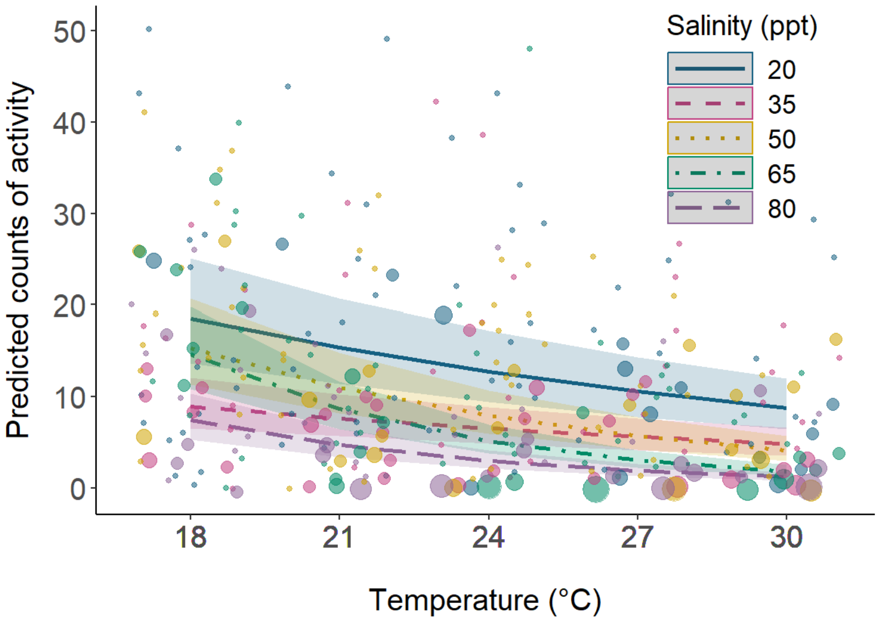

Fig. S3. Copepod activity declines with increasing salinity and temperature. Predicted counts of activity are from a generalized linear mixed model with fixed effects of salinity, temperature, and an interaction between the two, and a random effect of individual. Colored ribbon around each line denotes the confidence interval for predictions at that salinity and temperature. Dots represent raw counts of activity, with size increasing with number of occurrences at that point.

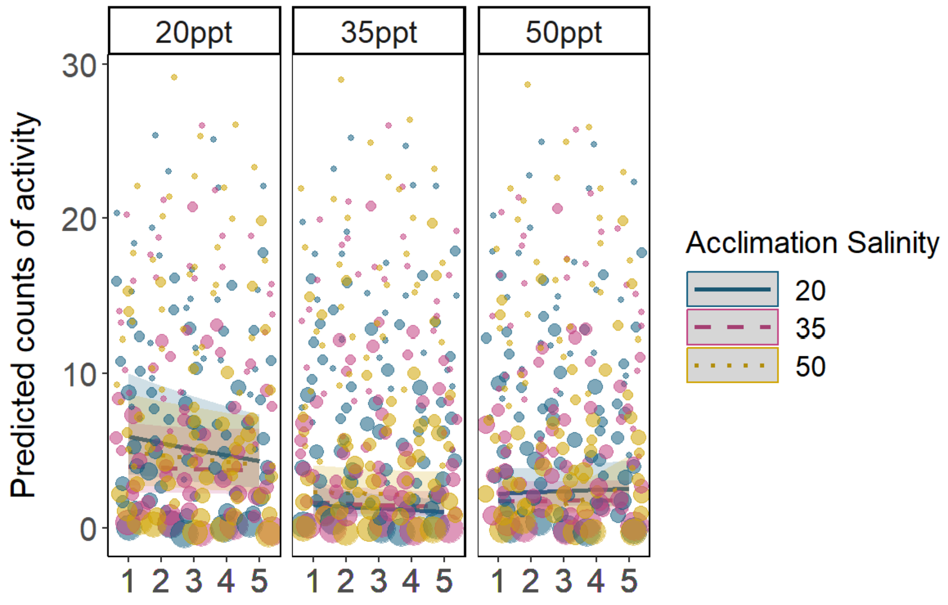

Fig. S4. Acute salinity transfer impacts rates of copepod activity. Predicted counts of activity are from a generalized linear mixed model with fixed effects of acclimation salinity, assay salinity, time after transfer, interactions between all fixed effects, and a random effect of individual. Colored ribbon around each line denotes the confidence interval for predictions at that salinity and temperature. Assay salinities are represented by plot facets. Dots represent raw counts of activity, with size increasing with number of occurrences at that point.

Journal of Experimental Biology • Supplementary information

Table S1. Summary of the statistical linear mixed effects model (with individual as a random factor) results testing for effects of chronic salinity acclimation, temperature, measurement interval, and their interaction on hourly copepod RMR.

Factor	Chi square value	Df	p-value
**Salinity**	**72.8715**	**4**	**5.616e-15**
Temperature	3.8032	1	0.051156
**Hour**	**18.3611**	**8**	**0.018677**
Salinity*Temperature	3.8546	4	0.426045
**Salinity*Hour**	**44.1159**	**28**	**0.027060**
**Temperature*Hour**	**23.9016**	**7**	**0.001186**
Salinity*Temperature*Hour	24.5297	28	0.653295

Significant terms are bolded. Measurements in this analysis were taken over the course of 9 hours and intervals (hour) consist of hour-long blocks. The model also included random effects of plate and copepod ID.

Table S2. Summary of the statistical linear mixed effects model (with individual as a random factor) results testing for effects of acute salinity transfer, temperature, measurement interval, and their interaction on hourly copepod RMR.

Factor	Chi square value	Df	p-value
**Acclimation salinity**	**71.3384**	**2**	**3.229e-16**
Assay salinity	5.7741	2	0.0557411
Temperature	2.3166	1	0.1279965
Hour	12.3208	8	0.1374554
**Acclimation salinity*Assay salinity**	**34.2080**	**2**	**6.755e-07**
**Acclimation salinity*Temperature**	**14.2569**	**2**	**0.0008020**
Assay salinity*Temperature	1.9763	2	0.3722603
**Acclimation salinity*Hour**	**90.9532**	**16**	**1.669e-12**
Assay salinity*Hour	6.4151	14	0.9549207
**Temperature*Hour**	**16.3106**	**7**	**0.0224252**
Acclimation salinity*Assay salinity*Temperature	6.2013	4	0.1846118
**Acclimation salinity*Assay salinity*Hour**	**60.4167**	**28**	**0.0003602**
Acclimation salinity*Temperature*Hour	20.8482	14	0.1055864
Assay salinity*Temperature*Hour	11.2698	14	0.6647226
Acclimation salinity*Assay salinity*Temperature*Hour	19.9473	28	0.8663773

Significant terms are bolded. Measurements in this analysis were taken over the course of 9 hours and intervals (hour) consist of hour-long blocks. The model also included random effects of plate and copepod ID.

Journal of Experimental Biology • Supplementary information
